# Local Control of Primary Dural Central Nervous System Lymphoma Achieved With Radiotherapy

**DOI:** 10.7759/cureus.15735

**Published:** 2021-06-18

**Authors:** Alexandra Vallejo, Iván D González, Eduardo Guerrero Lizcano, Oscar Forero, Leonardo Enciso

**Affiliations:** 1 Radiation Oncology, Instituto Nacional de Cancerología, Bogotá, COL; 2 Radiology, Instituto Nacional de Cancerología/Universidad Militar Nueva Granada, Bogotá, COL; 3 Hematology, National Cancer Institute, Bogotá, COL

**Keywords:** primary dural lymphoma, marginal zone lymphoma, radiation therapy, chemotherapy, primary central nervous system lymphoma

## Abstract

Primary central nervous system lymphomas (PCNSL) are extranodal non-Hodgkin's lymphomas (NHL) confined to the brain, leptomeninges, eyes, or spinal cord. Primary leptomeningeal lymphoma (PLML), without parenchymal involvement, synchronous cerebrospinal, or systemic disease is rare. The estimated incidence of PLML is 7% of all PCNSL, which in turn accounts for about 2% of all primary brain tumors and 0.8% of all lymphomas.

The incidence of PCNSL in Western countries is approximately five cases per million inhabitants per year, and less than 5% of all primary tumors of the central nervous system (CNS), although it is worth mentioning that the incidence seems to be increasing. The largest series of cases reported in the medical literature collect information from no more than nine patients; in these series, the median age at diagnosis is 57 years; in general, all patients present with cerebrospinal fluid alteration, and the median overall survival rate is close to eight months.

With our case series, we aim at sharing the experience of four patients diagnosed and treated at the National Cancer Institute between 2010 and 2020, establishing a correlation of the clinical, imaging, and histopathological presentation, the response to treatment based on radiotherapy and chemotherapy, and the clinical outcomes reported in the medical records.

## Introduction

Primary central nervous system lymphoma (PCNSL) is a type of extranodal non-Hodgkin's lymphoma (NHL) confined to the brain, leptomeninges, eyes, or spinal cord [1]. Primary leptomeningeal lymphoma (PLML) without synchronous cerebrospinal parenchymal involvement or systemic disease is rare; the estimated incidence of PLML is 7% of all PCNSL, making up for approximately 2% of all primary brain tumors and 0.8% of all lymphomas [2].

The incidence of PCNSL in Western countries is close to five cases per million people per year and accounts for less than 5% of all primary central nervous system (CNS) tumors, although it should be noted that the incidence of PCNSL seems to increase [3]. In this regard, few reports or case series have been published in the medical literature, and information from no more than nine patients has been collected among the largest series published so far. In this series, the median age at the time of diagnosis was 57 years; all patients had abnormal cerebrospinal fluid and the median survival rate was eight months [4].

Radiation therapy played a key role in the treatment of PCNSL until the late 1970s. External radiation therapy following surgical resection used to be the exclusive standard treatment for PCNSL. However, the results in patients treated exclusively with this treatment modality were generally less favorable as compared to current multimodal approaches, and they barely reached five-year survival rates mostly below 10% [5]. The role of radiotherapy in the treatment of PCNSL has recently changed, due to the increasing use of systemic chemotherapy [6].

PCNSLs may have a lower response to radiation therapy compared to other extranodal lymphomas. Historically, whole-brain irradiation (WBRT) was used to treat patients with PCNSL, as tumor cells often invade the normal brain located a few centimeters from the tumor mass [5]. Unfortunately, the combined regimen of radiation therapy and chemotherapy is associated with a high incidence of severe neurotoxicity, particularly in older patients [7]. Although omitting a sufficient dose of holoencephalic radiation therapy reduces neurological toxicity, it also results in a higher rate of relapses [8]. Technological progress has made it possible to propose using radiation therapy for the compromised field (involved-field radiation therapy, IFRT) instead of WBRT, by using magnetic resonance imaging (MRI) fusion for delimiting treatment volumes, thus, achieving relatively low irradiation of normal tissue as well as low radiation dose to the entire brain tissue. One of the most widely used RT regimens is to deliver low radiation doses (18-24 Gy) [9].

Primary dural lymphoma is a rare type of intracranial lymphoma, almost always exhibiting a histological variant and marginal zone immunophenotype, which often remains localized and is therefore potentially curable by using external radiation therapy (EBRT) exclusively [10]. A combination of WBRT/IFRT, or even low doses of IFRT delivered exclusively have shown to provide excellent local control. This approach is potentially curative and may possibly have lower clinically significant neurotoxicity rates [10].

Technical considerations of radiotherapy treatment

A simulation CT scan with or without contrast is performed, obtaining cuts of 3 mm from the apex to the level of C2; patients are positioned supine and immobilized with a thermoplastic facial mask, to achieve accurate and reproducible radiotherapy. In the case of having MRIs, they are fused to determine the volume to be treated more accurately [10]. Delimitation of volumes for the treatment plan (PTV) is set by adding a margin-locus of 0.5 cm to the CTV (tumor Volume clinical) to counteract the uncertainties of patient positioning during treatment, and the CTV is set up by adding 0.5 cm to the GTV (Volume macroscopic tumor), which is delimited based on the evidence of macroscopic disease visible on diagnostic images. Regarding treatment plan assessment, minimum coverage of 95% of the PTV with at least 95% of the total prescription dose was defined to be able to approve the treatment plan. The mean doses of IFRT and total RT were 12 Gy (range, 9-30 Gy) and 30 Gy (range, 30-42 Gy), respectively [10].

## Case presentation

Case #1

A 35-year-old woman in March 2017 started having episodes of intense headache; a tumor lesion became evident when a brain MRI was performed; a biopsy was taken on July 07, 2017, initially reporting a sickle tumor (apparently a meningioma), and the tumor was resected on August 27, 2017, whose pathology reported a sickle tumor, a hematolymphoid-like lesion to be classified by immunohistochemistry, and a surgical complication with a CSF fistula occurred. She was referred to the hematology group, which started management as a lymphoproliferative syndrome.

**Figure 1:**
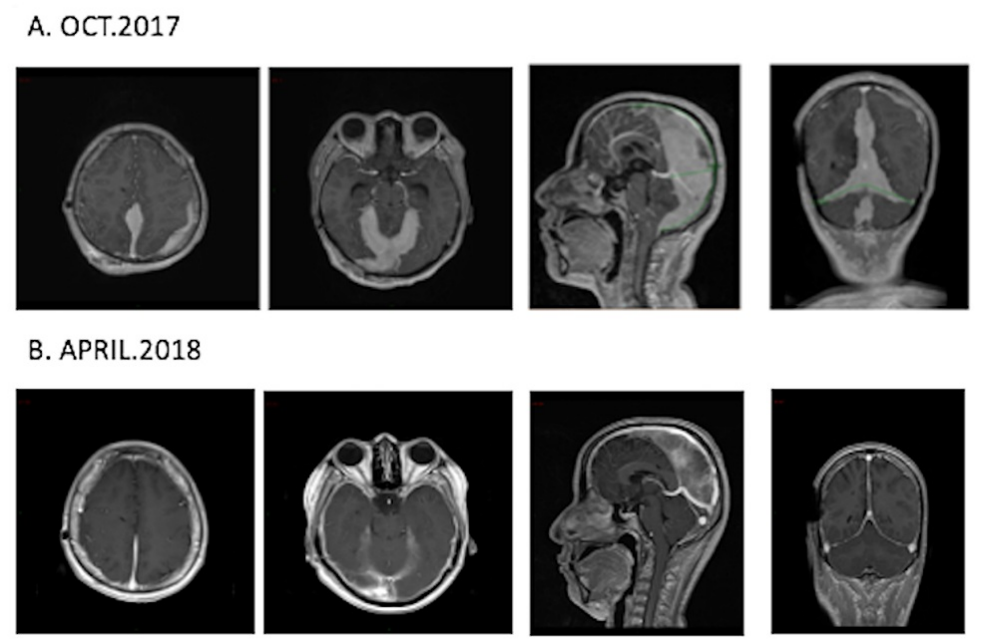
Comparative brain MRI (A) Brain MRI (October 2017): extensive infiltrative involvement of multiple portions of the dura mater, particularly in supra and infratentorial interhemispheric regions and in the tentorium. Additional lesions at the diploe level are present, highly suggestive of a hematogenous neoplasm with extranodal involvement. (B) Brain MRI (April 2018): radiological evolution of primary CNS lymphoma following external radiotherapy from December 11, 2017, to January 03, 2018: WBRT 36 Gy/2Gy with evidence of dura mater infiltration compatible with partial response, with signs of neoplastic infiltration persistence in the tentorium and in the posterior region of the brain sickle.

A brain MRI was performed in October 2017 (Figure 1), which evidenced extensive infiltrative involvement of multiple portions of the dura mater, particularly in supra and infratentorial interhemispheric regions and in the tentorium, which, given their characteristics and associated lesions in diploe, were highly suggestive of ovoid hematogenous neoplasia with extranodal involvement.

Pathology Study of the Brain, Sickle, and Lesion on October 23, 2017

**Figure 2:**
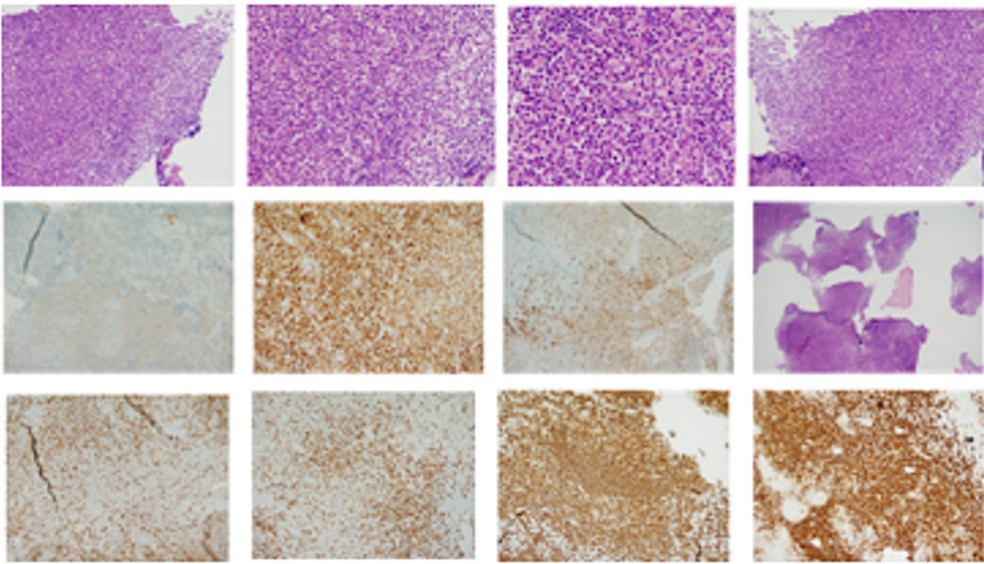
Pathology: immunohistochemical CD20 (+), CD79A(+), CD38(+), CD138(+), LMO2 (focal), CD10 (-), CD56 (-), EMA (-), and RP (-); CD30-positive. The Ki67 proliferation index is 60%.

The study reports a mature B-cell neoplasm with an extensive plasmacytic component: an immunohistochemical study was performed, showing diffuse positivity for CD20, CD79A, CD38, CD138, LMO2 (focal), while there is negativity for CD10, CD56, EMA, and RP; CD30-positive, scattered immunoblastic-looking cells are frequently observed without LMP1 expression. A lower proportion of the T-cell population is immunoreactive for CD3 and CD5. The light chains show a predominance of Kappa over Lambda. The Ki67 proliferation index is 60% (Figure 2).

The patient started exhibiting clinical deterioration with right hemiparesis, muscle strength 3/5, and ataxic gait; it was decided to hospitalize her with anticonvulsant management and dexamethasone 32 mg EV bolus and continuous 8 mg every eight hours for 14 days and subsequent gradual decrease. Evaluation by ophthalmology without evidence of retinal lesions in papilla or anterior segment; a new contrasted brain MRI was performed on December 01, 2017, with partial response of the primary lymphoma of the CNS, a decrease of approximately 40% in the volume of the dural infiltrative lesion, which compromises the posterior aspect of the interhemispheric sickle and the tentorium. There was a decrease in vasogenic edema predominantly in the right parietal and occipital lobes and complete resolution of the edema in the cerebellar vermis. Areas of vasogenic edema and gliosis persist, predominantly over the right parietal and occipital lobes, showing a decrease when compared to the previous.

**Figure 3:**
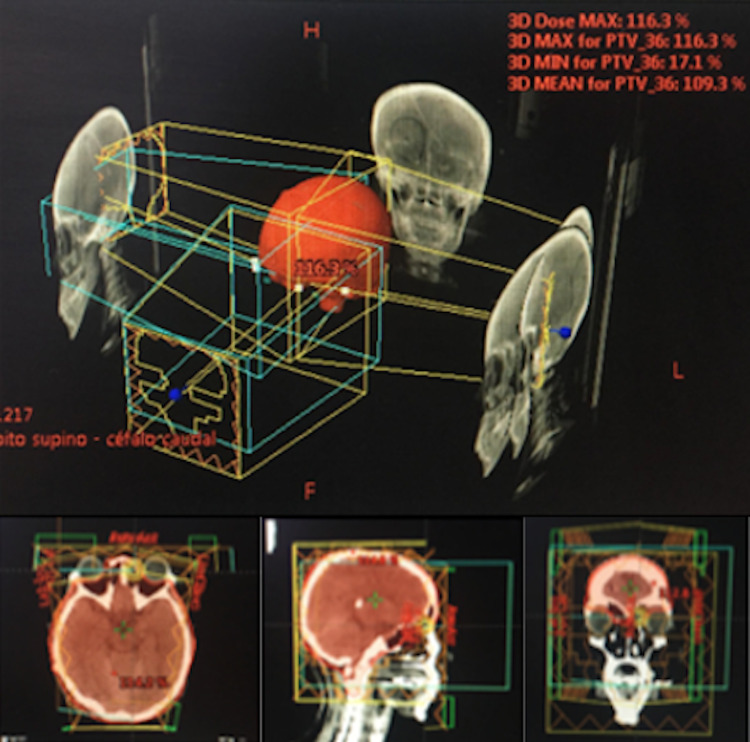
Treatment with conventional external radiotherapy holoencephalic technique 3DCRT technique external radiation therapy performed at the holoencephalic level (WBRT) from December 11, 2017, to January 03, 2018: a total dose of 36 Gy in 2Gy fractionation.

Given the partial response, the patient was considered to have primary CNS lymphoma with marginal histology classified as low risk according to ELSG:1 point - MSKCC - PSCNSL: 1 point and treatment with conventional external radiotherapy holoencephalic technique with a total dose of 3600 cGy in 200 cGy fractionation was decided. This treatment ended on January 03, 2018. The simulation CT was fused with previous brain MRI with gadolinium, to carry out an adequate and more accurate delimitation (Figure 3).

The patient suffered from fatigue, occasional headaches, and nausea during treatment, but did not require stopping treatment.

Follow-up brain MRIs showed partial response versus treatment-related findings. The PET-CT taken on December 4, 2019, reported that the study was negative for hyperglycolytic lesions of metabolically active lymphomatous infiltrative order. Her hemogram at the time of the last clinical evaluation was normal, and her physical examination showed no alteration, with 5/5 symmetrical muscle strength and preserved sensitivity without signs of meningeal irritation.

Case #2

A 46-year-old woman, who on November 16, 2010 consulted for a medical condition of six months of evolution that began with a decrease in strength of the right lower limb and headache. A brain CT was taken, which showed an acute left frontotemporal subdural hematoma, and she was taken to surgery for drainage on March 29, 2010. However, the patient reported that she continued to have dizziness, so an MRI study was ordered, which showed a subacute subdural collection of temporal predominance, with a thickness of 18 mm and a compressive effect on adjacent parenchyma. She was taken again to surgery, where no bleeding but tumor tissue of subdural origin without infiltrating the cortex was found, recalling a meningioma or a plasmocyte lesion, so partial resection was performed.

The initial pathology reported proliferation of plasma cells behavior to be defined; the immunohistochemistry reported an inflammatory pseudotumor (plasma cell granuloma) with CD38 (+) but CD56 (−) cells with normal Kappa/Lambda distribution.

Complete surgical resection was performed and the histopathological study reported an astrocytoma. Control images were taken showing a left frontotemporal extra-axial lesion, which due to its topographic characteristics was compatible with a meningioma or a hemangiopericytoma.

**Figure 4:**
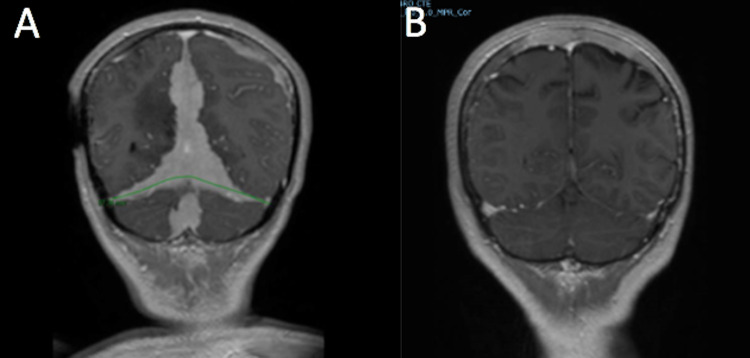
Comparative brain MRI (A) Brain MRI (performed in March 2010): subacute frontotemporal subdural collection, left anterolateral with a temporal predominance, with a maximum thickness of 18 mm with a compressive effect on adjacent parenchyma and asymmetry of the supratentorial ventricular system. (B) Brain MRI (performed in October 2010): persistence of diffuse signal alteration of the bone marrow in the calvarium, with a predominance in parietal regions.

A study with a contrasted brain CT-scan was performed on August 17, 2010 (Figure 4) showing an extra-axial frontotemporal left lesion of 52 × 17 mm^2^. The contrast medium was captured intensely and evenly, without producing changes in the underlying bone, but producing a mild collapse of the anterior horn of the lateral ventricle and left subfalcine herniation, with the remaining of the ventricular system with normal morphology and volume.

**Figure 5:**
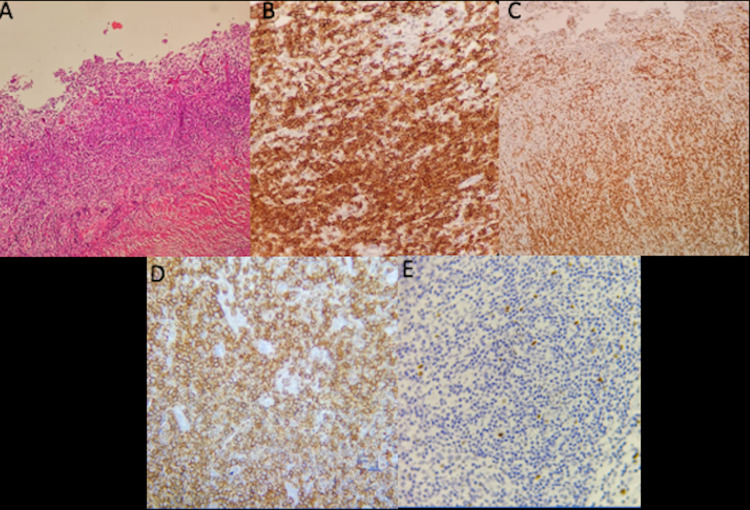
Pathology and immunohistochemistry (A) H&E, (B) CD20, (C) BCL2, (D) CD43, and (E) Ki67.

The institutional review of the pathology reported a CD20 (+) small cell non-Hodgkin B lymphoma, weak Bcl2-CD43 co-expression, low Ki67 kappa monotypicity, and negativity for CD23, Cyclin, CD10, Lambda, and CD3 (Figure 5).

**Figure 6:**
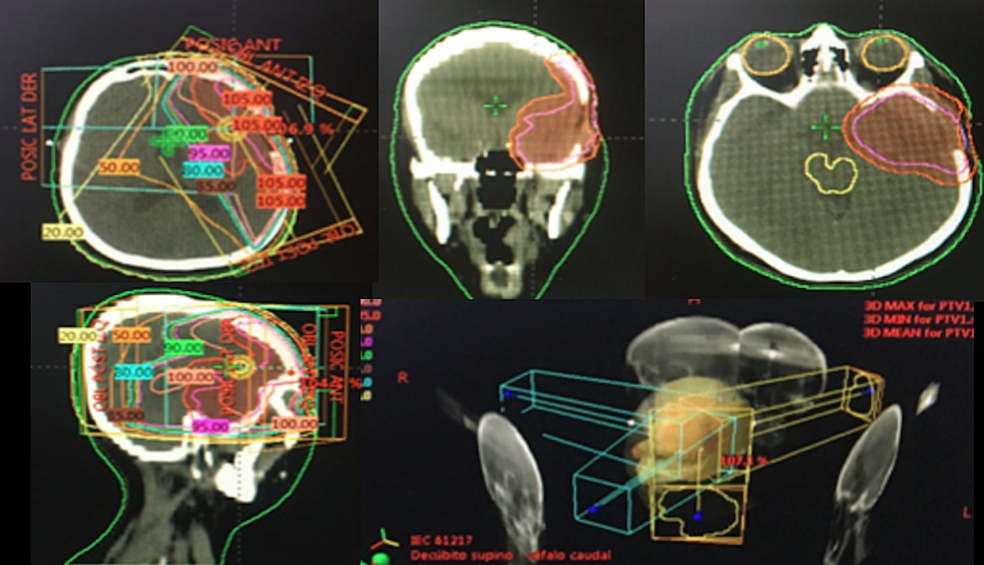
Exclusive external radiotherapy with curative intention; a conformational technique for a total dose of 4400 cGy in 200 cGy fractionation

Therefore, the patient was hospitalized for treatment with anticonvulsants and Dexamethasone 8 mg every eight hours for seven days, without alterations to the clinical and neurological evaluation. The case was presented at a multidisciplinary meeting and management with exclusive external radiotherapy with curative intention was considered; a conformational technique for a total dose of 4400 cGy in 200 cGy fractionation (Figure 6).

The patient underwent clinical and imaging follow-up with an adequate response to treatment, with a normal physical examination and PET-CT in September 2018, which reported negative for hypermetabolic lesions, particularly in the skull or brain. In October 2019, the patient manifested transient global amnesia, with a control MRI reporting persistence of diffuse alteration of the bone marrow signal in the calvaria with a predominance in parietal regions, which was classified as a complete response.

The patient is currently undergoing neuropsychological testing studies aimed at finding any other deficits and guide rehabilitation maneuvers, considering cognitive changes as secondary to radiotherapy treatment.

Case #3

**Figure 7:**
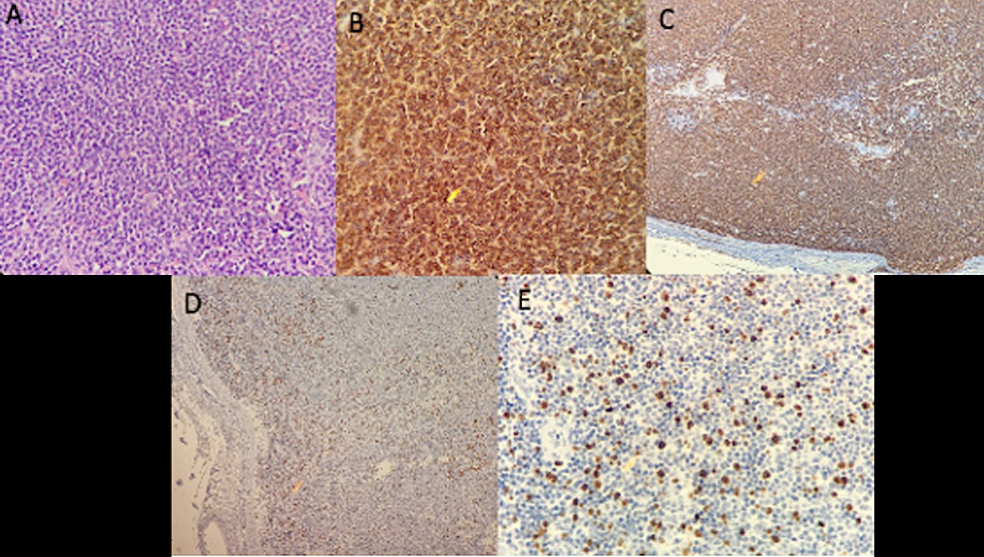
Pathology and immunohistochemistry (A) H&E, (B) CD20 – 40×, (C) BCL2 – 10×, (D) Ki67 – 10×, (E) Ki67 – 40×.

A 55-year-old female patient with a medical condition that began in January 2017 with swelling of progressive growth in the left cervical region, initially not painful, for which a biopsy was performed reporting an atypical lymphoid infiltrate, with immunohistochemistry compatible with a small-cell non-Hodgkin B lymphoma, probably marginal (Figure 7). CT images showed leptomeningeal, cerebral, and splenic involvement. At the time of evaluation, the patient presented an increase in the volume of the cervical mass associated with pain, night fever, diaphoresis, and weight loss.

**Figure 8:**
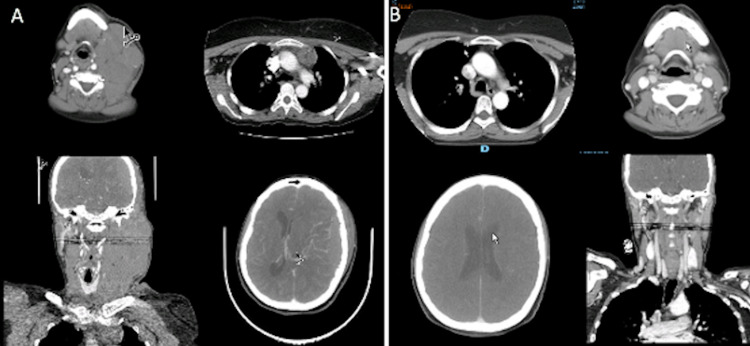
Comparative CT head and neck and brain MRI (A) CT of the neck and chest (October 2017): a mass with a neoplastic appearance occupying the left hemicneck, extending towards the cranial vault, and compromising the epidural space, with a mass effect on the brain parenchyma and subfalcine herniation. Prevascular and bilateral axillary lymph node involvement. Multiple adenomegalies in the right hemicneck. (B) Contrast brain MRI (September 2018): little residual-like dural enhancement in the left parietal location with no evidence of lesions suggesting residue or tumor recurrence. When compared to the initial study on October 31, 2017, the patient is considered to have a complete response.

The brain MRI performed on October 1, 2017, showed an extensive bilateral cervical lymph node tumor disease, predominantly on the left side, attributable to the diagnosis of lymphoma with greater involvement of the parapharyngeal and left parotid spaces. An extra-axial solid mass with left hemispheric intracranial and extra-cranial involvement, attributable to tumor infiltration by lymphoma, which exerts a significant compressive effect on the adjacent brain parenchyma, with subfalcine herniation on the right and descending uncal herniation (Figure 8).

The patient was hospitalized and received a pre-phase with high doses of dexamethasone. The follow-up brain imaging showed a 70% decrease in tumor volume. Treatment with chemotherapy was continued: R-CHOP scheme with intolerance due to severe toxicity, for which it was decided to change chemotherapy to R Bendamustine (six cycles) between December 2017 and April 2018, with a very good response including nodal involvement by image tracing. However, considering the initial lumpy images with cranial involvement, consolidation was considered with 3DCRT Holoencephalic external radiation therapy in fractionation of 2 Gy up to a total dose of 30 Gy, which ended on August 2, 2018. PET CT in 2018 did not report evidence of hypermetabolic lesions suggestive of tumor involvement by lymphoma.

By-annual clinical and imaging follow-ups show a physical examination without alterations suggesting recurrence of the lymphoproliferative disease, and resolution of the tumor lesions. The patient is considered to have a complete response.

Case #4

**Figure 9:**
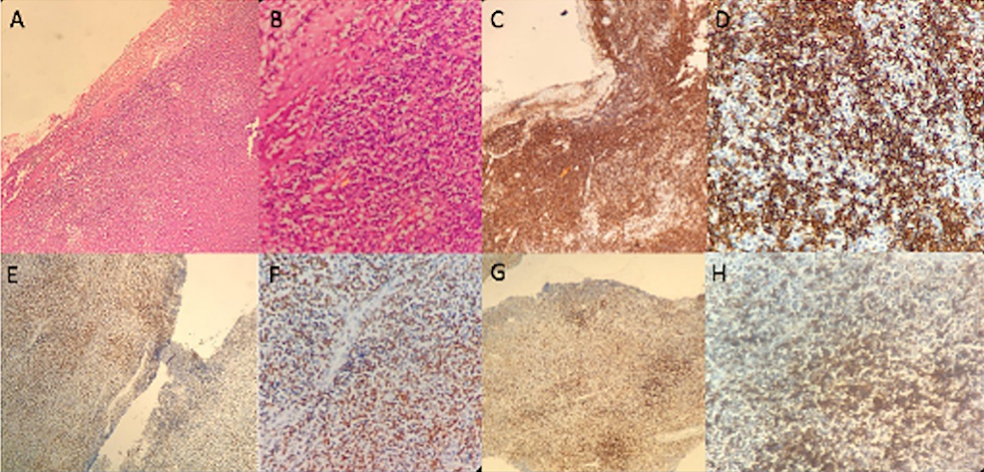
Pathology and immunohistochemistry (A) H&E -10×, (B) H&E – 40×, (C) CD20 – 10×, (D) CD20 – 40×, (E) BLC2 × 10×, (F) BLC2 × 40×, (G) CD43 × 10×, (H) CD43 × 40×.

A 34-year-old male patient was presented with right temporal sentinel headache and seizures in March 2017. A CSF study was performed with normal results, and images that evidenced thickening and hyper-uptake of the meninges in the right frontoparietal temporal region, extending to the cortical grooves; a biopsy was taken, reporting meninges with non-Hodgkin B lymphoma compatible with extranodal marginal lymphoma; IHC with positivity for CD20, CD43 and BCL-2, KI 67 <5%, and negativity for CD10, BCL-6, cyclin D1 and CD56 (Figure 9).

In the brain MRI image of September 2017, surgical changes in the approach by right supraorbital frontal craniotomy and meningeal thickening area were observed. The rest of the extension studies did not indicate extracranial involvement. The case was presented in a multidisciplinary meeting considering that the best therapeutic alternative was to perform 3DCRT technique external radiotherapy at the holoencephalic level with subsequent reinforcement of the macroscopic lesion, with a total dose of 24 Gy in fractionation of 1.5 Gy and sequential boost up to 36 Gy, which ended in September 2017. During radiotherapy, the patient presented headaches and nausea that were controlled with symptomatic treatment but did not require stopping treatment.

A follow-up brain MRI image from January 2018 revealed a zone of meningeal nodular thickening adjacent to the craniotomy, which may correspond to postsurgical fibrotic changes.

In clinical and imaging follow-up, a physical examination reported no alteration and was classified as an extranodal marginal lymphoma of the dura mater, with a complete post-radiotherapy response.

In the brain MRI of October 2, 2020 compared to the 2019 brain MRI, a new nodular thickening of the meninx appeared, which lies on the greater wing of the sphenoid, with approximately 11 mm thick × 22 mm of base extension that enhances with contrast medium. Postsurgical changes of right anterior frontal craniotomy, gliosis, and encephalomalacia of the anterior pole of the right frontal lobe were observed.

## Discussion

Leptomeningeal lymphoma was first reported in 1997 by Kumar et al. [11], as an indolent type B cell lymphoma mainly of extranodal origin in the marginal zone, which occurred mainly in middle-aged adult women, its main characteristic being the absence of systemic involvement or lymphomatous infiltration to the central nervous system. Since no lymphoid tissue is normally found in the dura mater, the pathogenesis of PLML has not been clearly described.

Most of these types of lymphomas are MALT type (lymphoid tissue associated with mucous membranes), lineage B with characteristics of cells of the marginal zone originating in lymphoid nests housed in the mucosal tissues. Some cases with other histological subtypes have been reported including follicular, Hodgkin's, and diffuse large cell B lymphomas. Such lymphomas can be epidural, dural, subdural, subarachnoid, or combinations of those types.

The incidence of PLML in developed countries is less than 5% of all primary CNS tumors, although the incidence appears to be increasing. It poses a diagnostic and therapeutic challenge since they are often initially confused with other types of pathologies such as meningiomas.

Many cases have been reported in the literature; however, these reviews have had a very varied focus, in most cases oriented towards histopathological characteristics rather than on the therapeutic approach. Given the hematolymphoid nature of this disease, treatment with chemotherapy has traditionally been preferred; our case series aims at sharing the experience with four patients diagnosed and successfully treated at the National Cancer Institute of Colombia between 2010 and 2020; we expose the correlation of clinical, imaging, and histopathological presentation, and express the diagnostic challenges of the described condition, as well as the response to treatment based on exclusive radiotherapy or radiotherapy added to chemotherapy.

The clinical outcomes reported in our patients demonstrate a successful response to the proposed therapy, with very good control of the disease in the long term. We consider that the series we present supports the concept that holoencephalic radiotherapy, with advanced radiotherapy techniques such as IFRT, achieves local control with follow-ups up to 10 years, and remains an option for proper curative treatment, without evidence of local or systemic relapse of the disease, and with a good toxicity profile.

## Conclusions

Primary lymphoma of the CNS poses both a diagnostic and therapeutic challenge; studies focused on the standardization of the total dose, fractionation, and radiotherapy technique are required, to obtain recovery with the least possible side effects; our series of cases illustrates the benefit of incorporating external radiotherapy as a fundamental tool for the treatment of patients in this spectrum of hematological pathology.

Finally, readers and potential researchers are invited to develop research protocols focused on radiotherapy, its impact on recovery, and possible short and long-term adverse effects, which will allow radiotherapy to be included as a foundation for the treatment of primary meningeal lymphoma.
